# Pan-Genomic Analysis Provides Insights into the Genomic Variation and Evolution of *Salmonella* Paratyphi A

**DOI:** 10.1371/journal.pone.0045346

**Published:** 2012-09-19

**Authors:** Weili Liang, Yongbing Zhao, Chunxia Chen, Xiaoying Cui, Jun Yu, Jingfa Xiao, Biao Kan

**Affiliations:** 1 State Key Laboratory for Infectious Disease Prevention and Control, and National Institute for Communicable Disease Control and Prevention, Chinese Center for Disease Control and Prevention. Beijing, People’s Republic of China; 2 CAS Key Laboratory of Genome Sciences and Information, Beijing Institute of Genomics, Chinese Academy of Sciences, Beijing, People’s Republic of China; 3 Graduate University of the Chinese Academy of Sciences, Beijing, People’s Republic of China; University of Lausanne, Switzerland

## Abstract

*Salmonella* Paratyphi A (*S*. Paratyphi A) is a highly adapted, human-specific pathogen that causes paratyphoid fever. Cases of paratyphoid fever have recently been increasing, and the disease is becoming a major public health concern, especially in Eastern and Southern Asia. To investigate the genomic variation and evolution of *S.* Paratyphi A, a pan-genomic analysis was performed on five newly sequenced *S.* Paratyphi A strains and two other reference strains. A whole genome comparison revealed that the seven genomes are collinear and that their organization is highly conserved. The high rate of substitutions in part of the core genome indicates that there are frequent homologous recombination events. Based on the changes in the pan-genome size and cluster number (both in the core functional genes and core pseudogenes), it can be inferred that the sharply increasing number of pseudogene clusters may have strong correlation with the inactivation of functional genes, and indicates that the *S.* Paratyphi A genome is being degraded.

## Introduction

*Salmonella* are important human and animal pathogens and include two species, *Salmonella bongori* (*S. bongori*) and *Salmonella enterica* (*S. enterica*). *S. enterica* are further classified into distinct subspecies, and include more than 2500 serovars [1]. Most serovars within *S. enterica* infect a broad range of host species and cause self-limiting gastroenteritis [2], [3]. However, a few serovars, including *S.* Typhi and *S.* Paratyphi A, B and C, only infect humans and elicit typhoid, paratyphoid or enteric fever, which are severe infections of the reticuloendothelial system with high rates of complications and mortality [4], [5]. The clinical features of paratyphoid fever resemble typhoid fever, but it is a relatively milder illness, and an accurate differential diagnosis relies on laboratory confirmation [6], [7].

*S.* Typhi-related typhoid fever is the most-studied enteric fever, and is responsible for an estimated 21,650,974 cases and 216,510 deaths annually worldwide, based on the latest burden estimation study [8]. It was generally believed that the number of paratyphoid fever cases ranged from 0.11 to 0.35 for every case of typhoid fever [8]. But during the past few decades, the *S.* Paratyphi A infection rates have been rising in Southeast Asia [9]. In Nepal, this specific serovar currently accounts for 30–50% of enteric fever illnesses [9]–[11]. In India, the incidence of *S.* Paratyphi A in enteric fever cases increased from 6.5% in 1994 to 44.9% in 1998 [12]. Moreover, more and more isolates display multidrug resistance and are associated with large outbreaks [10], [13]. Increases in the proportion, as well as the absolute number, of *S.* Paratyphi A infections have also been reported widely in China, and *S.* Paratyphi A has become the most prominent serovar in most endemic provinces, such as Guangxi, Zhejiang, Guizhou, Yunnan, and Jiangsu [14]–[19]. The increasing trend in *S.* Paratyphi A infection and the emergence and worldwide spread of multi-drug resistant strains make it a major public health concern due to the lack of an effective vaccine and the difficulty treating these infections with conventional drugs.

Information about the whole genome sequence and genes present in *S.* Paratyphi A will help to understand the strain’s evolution and pathogenesis, and will reveal more specific targets for drug design and vaccine development. Currently, six complete genomic sequences for the enteric fever pathogens are available at GenBank, two for *S.* Typhi isolates (clinical isolate CT18 and laboratory strain Ty2) [20], [21], two for *S.* Paratyphi A strains (clinical isolate AKU-12601 and laboratory strain ATCC9150) [22], [23], and one each for the *S.* Paratyphi B strain SPB7 and *S.* Paratyphi C strain RKS4594 [24]. Although Falush and Didelot’s study [25], [26] showed that S. Typhi and *S.* Paratyphi A are of different lineages, the whole genome comparative analyses indicated that the *S.* Typhi and *S.* Paratyphi A strains are closely related at the DNA level [22], [23], while *S.* Paratyphi C is more closely related to *S.* Choleraesuis than to *S.* Typhi or *S.* Paratyphi A [24]. A key feature of these human-restricted enteric fever agents is their higher accumulation of pseudogenes compared to the host-generalist serovars, which is thought to be due to a combination of adaptation to the host and genetic drift associated with population bottlenecks encountered during the course of their adaptation to the new niche [20], [22], [27]. Some pseudogenes are shared between these serovars [22]. It has been proposed that pseudogenes, fimbrial operons, virulence plasmids, lysogenic phages and genes belonging to *Salmonella* pathogenicity islands (SPI) are involved in conferring host specificity and restricting the host range [28], [29]. In particular, pseudogenes are thought to play a key role in the host adaptation of the typhoid agents [21], [22].

It was reported that *S.* Paratyphi A isolates from different geographical regions in Asia displayed genetic diversity by pulsed-field gel electrophoresis (PFGE) [30] and the same phenomenon was noted in China [31]–[33]. To assess the natural variation of the different clones involved in the recent paratyphoid fever epidemics and to identify the individual changes in these clones, we sequenced five epidemic *S.* Paratyphi A strains isolated in China by Illumina/Solexa sequencing and performed a comparative genomic analysis between these newly sequenced *S.* Paratyphi A strains and two reference strains, ATCC9150 and AKU-12601. The five epidemic strains were isolated from different high-incidence endemic provinces at different times, and displayed different PFGE patterns. Our analysis clarifies the *S*. Paratyphi A genomic evolution profile, which will improve our knowledge about the dynamic variation in *S*. Paratyphi A.

## Results and Discussion

### General Features of Sequenced Genomes

After assembling the reads from the Solexa sequencer, 24 to 30 scaffolds were available for the five epidemic strains. All of the gaps in the scaffolds output from the SOAPdenovo software program were filled by fishing out related reads manually and by PCR experiments. The scaffold N50 values of the assembly results ranged from 300 kb to 313 kb, and the scaffold N90 values were about 112 kb. The lengths of the total scaffolds of each newly sequenced strain were about 4.5 Mb, which is the same as the two reference strains, AKU_12601 (NC_011147) and ATCC_9150 (NC_006511) (**Table 1**). As for the protein coding genes, the functional gene numbers of the GXS2268 (AFYX00000000), GZ9A00052 (AFYW00000000), JX05-19 (AFYY00000000), YN09620 (AFYZ00000000), and ZJ98-53 (AFZA00000000) epidemic strains, and the two reference strains (AKU-12601 [22]and ATCC9150 [23]) were 4052, 4125, 4122, 4059, 4047, 4161 and 4159, respectively, while the pseudogene numbers were 279, 236, 244, 226, 262, 188 and 211, respectively. To make subsequent comparisons consistent, the genome sequences of the two reference strains, AKU_12601 and ATCC_9150, were re-annotated, and the new predicted protein coding genes in the two reference strains are listed in **Table S1**.

**Table 1:** The results of genome sequencing and assembly.

	GXS2268	GZ9A00052	JX05-19	YN09620	ZJ98-53
High-quality reads pair number	4,640,879	3,057,990	3,068,605	3,834,202	3,158,039
Scaffold N50 (bp)	313,753	313,777	313,564	313,195	299,832
Scaffold N90 (bp)	112,704	112,666	112,618	112,449	112,448
Scaffold Number	25	30	27	24	24
GC Content (%)	52.17	52.16	52.16	52.18	52.2
Total Scaffold Length	4,525,299	4,555,645	4,556,399	4,490,100	4,516,726

### The Pan-genomic Analysis of *S.* Paratyphi A

#### *S*. Paratyphi A has a highly conserved genomic structure

**Figure 1:**
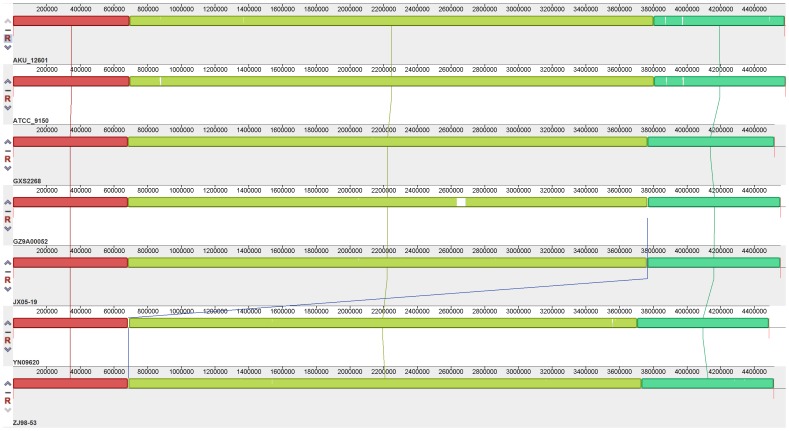
A comparison of the genomic structures of all seven strains. The structures shown from top to bottom are for GXS2268, GZ9A00052, JX05-19, YN09620, ZJ98-53, AKU_1260 and ATCC_9150. The same colors represent homologous fragments identified by the Mauve program.

After all scaffolds of each strain were connected into a pseudochromosome, these five pseudochromosomes were aligned with the other two reference chromosomes (**Figure 1** **)**. From the whole genome alignment results, it was clear that all seven strains kept highly consistent synteny in the structure of their genomes. Excluding the linker sequences for each scaffold, the cumulative lengths of the conserved regions was about 4.38 Mb, which accounts for 95.5%∼97.5% of the pseudochromosome or chromosome.

#### The pan-genomic composition of functional genes in *S*. Paratyphi A is also highly conserved

The pan-genome denotes the gene repository of a species, and it includes three parts: the core genome shared by all strains, the dispensable genome shared by two or more (but not all) strains, and the strain-specific genome [34], [35]. To study the pan-genomic composition of *S*. Paratyphi A, we categorized all 28725 functional genes from the seven strains into 4252 orthologous clusters (**Table S2**), among which 3720 clusters were considered to be core clusters, 465 clusters were dispensable clusters (shared by two to six strains) and 67 were strain-specific clusters. The core clusters accounted for 87.5% of the total 4252 clusters. Among the 465 dispensable orthologous clusters, 351 clusters were shared by six strains. Compared to the high percentage of core clusters in the seven *S*. Paratyphi A strains, *Escherichia coli* (*E*. *coli*) strains, which are in the *Escherichia* genus within the same *Enterobacteriaceae* family, display a much lower percentage, with 17 strains sharing only 2344 conserved genes (46.7% of the total 5020 clusters) [36]. *Streptococcus agalactiae* (*S. agalactiae*)[also known as Group B *Streptococcus* (GBS)], a pathogen with a long-distance evolutionary relationship to *S*. Paratyphi A, shares 1486 conserved genes (57.8% of total 2571 genes) in eight strains [34]. Hence, it can be inferred that *S*. Paratyphi A is a very conservative pathogen with regard to its pan-genomic composition.

#### *S*. Paratyphi A has a closed pan-genome of functional genes

**Figure 2:**
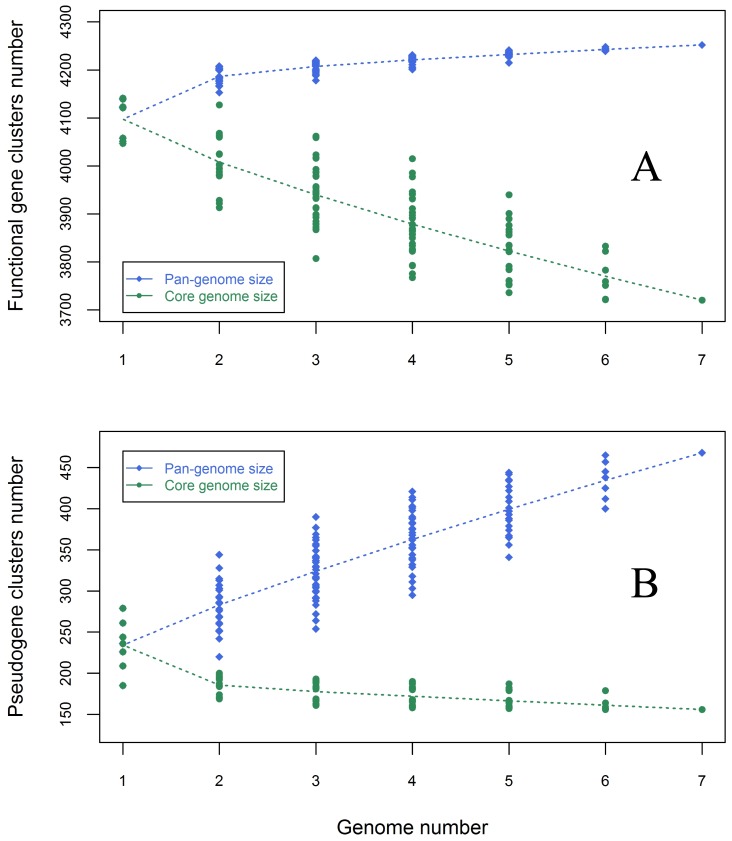
The pan-genomic profile curves for both functional gene clusters and pseudogene clusters. A) The blue diamonds denote the functional pan-genome size for each combination and the blue dotted line denotes the relationship between the genome number and functional pan-genome size. The green dots denote the core functional clusters for each combination and the green dotted line denotes the relationship between the genome number and the core functional cluster number. B) The blue diamonds denote the pseudo pan-genome size for each combination, and the blue dotted line denotes the relationship between the genome number and the pseudo pan-genome size. The green dots denote the core pseudogene clusters for each combination and the green dotted line denotes the relationship between the genome number and the core pseudogene cluster number.

Usually, the number of pan-genome clusters and core genome clusters depends on how many different strains’ genomes are being considered in the analysis. The power law regression model allowed us to extrapolate for a certain group of strains to predict whether the number of different gene clusters found in *S*. Paratyphi A is finite (a closed pan-genome), or unlimited (an open pan-genome). In the past studies on bacterial pan-genomes, some strains have been found to have an open pan-genome of functional genes, such as *S. agalactiae*, *S. pneumoniae*, *E. coli*, *B. cereus*, and *P. marinus* [34], [36]–[39], while others have a closed pan-genome of functional genes, such as *S. aureus*, *S. pyogenes*, *Ureaplasma urealyticum* (*UU*), and *Bacillus anthracis* (*B. anthracis*) [38]. To understand the relationship between the *S*. Paratyphi A pan-genome size or the core functional gene cluster number and the number of strains, we calculated the pan-genome size of functional genes and the core functional gene cluster number for n (n = 1, 2…7) *S*. Paratyphi A strains. When we calculated the number of core functional gene clusters shared by n strains, the number of core functional gene clusters shared by more than n strains would not be included. At the same time, to avoid random bias, when we calculated the n strains’ pan-genome size for functional genes or the core functional gene cluster number, all combinations of the seven strains were analyzed, and their mean value was adopted to fit the core genome clusters curve and pan-genome clusters curve (**Figure 2A**).

From this calculation, it was apparent that the pan-genome curve converges rapidly, and there are very few increases in the functional gene cluster number after the strain number (n) >5, which indicates that there are few new genes that will be identified if more strains are sequenced. We found that the pan-genome size of *S*. Paratyphi A fits Heaps law well. The mathematical function of the pan-genome size model is as follows:
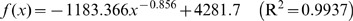



In the function, x represents the number of sequenced genomes. By using this model, we determined that there would be about 4282 functional gene clusters (4280∼4284, 95% confidence) in *S*. Paratyphi A strains.

Exchanging genetic material with the outside world is typically the driving force of evolution for bacteria, but the converged pan-genome size of functional genes implies that *S*. Paratyphi A imports few new genes from other species. In general, new genes can be originated from different mechanisms, such as duplication of existing sequences and then diversification, importation of new genetic material from other species, and so on. Nevertheless, horizontal gene transfer is considered to be the main evolutionary process that enables pathogens to adapt to a changing niche [40]–[43]. In the three parts of the functional pan-genome, the strain-specific genes are mainly newly acquired genes. In our seven analyzed *S*. Paratyphi A strains, only 67 out of 4252 clusters (1.6%) were strain-specific genes, while in *S. agalactiae* strains, 358 of 2571 clusters (13.9%) [34] were unique genes for that strain. In *E. coli*, about 300 new strain-specific genes are discovered when a new genome is sequenced [36]. The low strain-specific gene rate of *S*. Paratyphi A indicates that it is deficient in gaining new genes, which may contribute to its conservative genomic structure and partly account for the high collinearity of the epidemic strains’ genomes.

From Figure 2A, it can be inferred that *S*. Paratyphi A has a closed pan-genome. However, it is possible that our conclusion about the closed pan-genome of *S*. Paratyphi A may be not definitive, because five of seven analyzed *S*. Paratyphi A strains were from the same geographic region. However, from the whole genome alignment results (**Figure 1** **)**, it is clear that the *S*. Paratyphi A genome structure is highly conserved among the five Chinese strains and two strains from other countries. The core functional genes comprise a fairly high proportion of the total gene pool, and the percentage of strain-specific genes is very small. Moreover, the pan-genome size converged very rapidly when the seven strains, including the two from other countries and the five newly sequenced Chinese strains, were analyzed together. Consequently, we can conclude that *S*. Paratyphi A has a closed pan-genome.

On the other hand, when the seven genomes were analyzed, the core functional gene cluster number was found to still be decreasing sharply, with no signs of convergence. Therefore, we deduced that the inner variation in *S*. Paratyphi A is the main evolutionary mechanism shaping its gene composition, and that importing new genes from outside genomes is not an important driving force in its evolution.

### Genomic Variation and Dynamics

#### SNPs in core functional gene clusters

From the whole genome alignment results and the composition of the pan-genome, it is easy to see that the core genome makes up the majority of the *S*. Paratyphi A genome. To assess the genomic variation, we first identified the SNPs present in the core genome. For this purpose, each core orthologous cluster was aligned. Because the ORFs in the gaps were not sequenced or were only partly sequenced, it was inevitable that some SNPs were missed, but the majority of the genome was included in the analysis. We also filtered the false positive indel events which were caused by discrepant initiation codons or partly sequenced ORFs.

**Figure 3:**
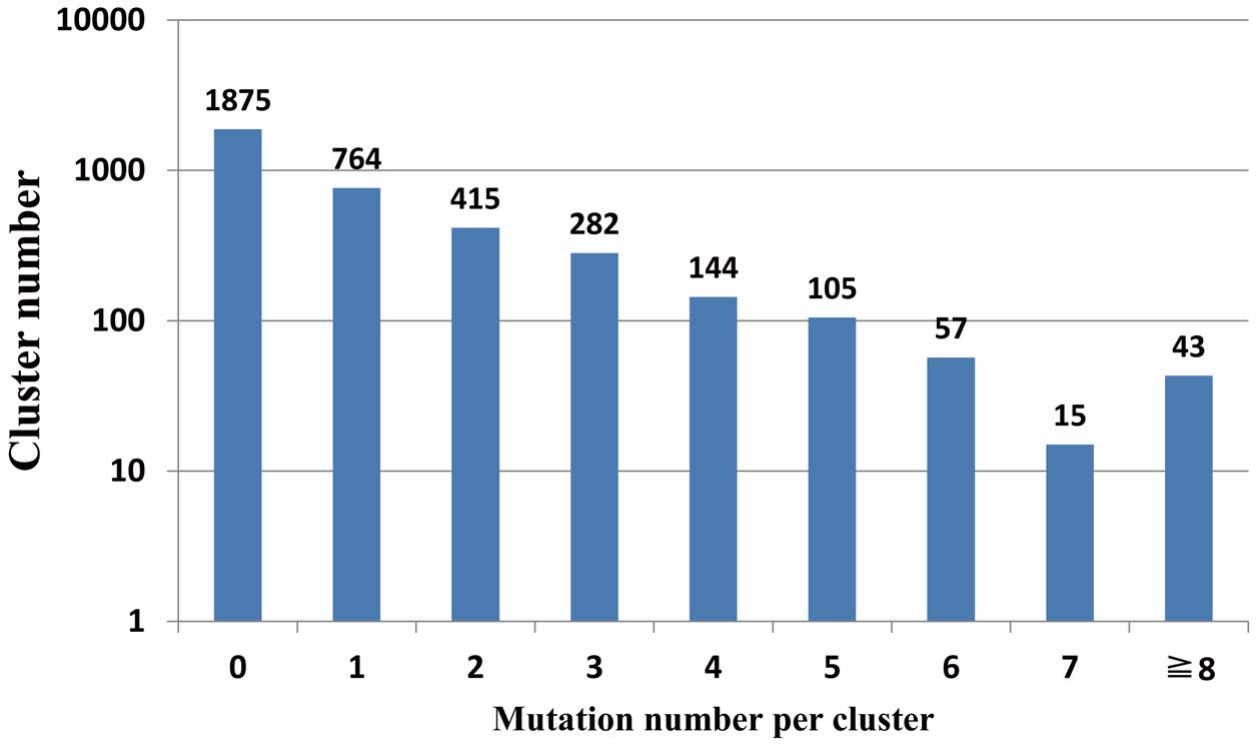
The distribution of the number of SNPs per cluster.

Among the selected 3712 core functional gene clusters (eight core clusters, in which one or more genes involved were only partly sequenced, were excluded), there were 90 clusters that involved indel events. In most of these clusters, the flanking sequences of the indel sites were consistent, while four clusters had some variations in their flanking sequences, so we inferred that these indel events may have been caused by recombination. In the other 86 clusters that had indel events without variations near the flanking regions of the indel sites, 22 clusters had short insertions at the end of the ORF, and in the remaining 64 clusters, there were 60 insertions and four deletions, and all of the 64 indel events occurred in the middle of the ORFs. Additionally, 4569 mutations were detected in 1824 clusters (of which 77 clusters also involved indel events), while the remaining 1875 clusters (50.5% of the total core gene clusters) had no SNPs **(** **Figure 3** **)**. Among the 1824 clusters with mutations, there were 529 clusters that suffered from a more than 0.3% mutation frequency. Moreover, 40 of these 529 clusters had a more than 1% mutation frequency. Besides these clusters, another nine clusters with a less than 0.3% frequency of mutation had accumulated no fewer than eight mutations (**Table S3**). In these clusters with a high mutation frequency or a high mutation number, most substitution events occurred in one or two genes rather than all or most of the genes in the clusters. It has previously been suggested that if three or more substitution events were present in a 1 kb region, they could be considered to be recombination events [44]. Using this definition, after we removed those clusters which included two or more genes from the same strain or partly sequenced genes, it was found that 380 genes from 336 core clusters had three or more substitutions in a 1 kb region. Moreover, in some of these genes, three or more substitutions occurred in a region smaller than 100 bp. This suggests that these substitutions may have been introduced by recombination, rather than occurring as natural mutations. In other words, 54 core genes (on average) in each strain might have undergone recombination events. A high homologous recombination rate was also found among *S. enterica* with the Multi Locus Sequence Typing (MLST) method by Vos et al [45]. At the same time, Didelot *et al* [46] found that *S.* Paratyphi A and Typhi have gone through a burst of recombination involving more than 100 recombination events, and this recombination has allowed the exchange of gene variants that are important for their adaptation to their common ecological niche. Among the 4569 substitutions in the 1824 clusters, 1434 substitutions may have been introduced by recombination events. The ratio between the number of substitutions introduced by recombination and mutation (r/m) is about 0.46, which may be slightly lower than the true ratio value due to the strict criteria used to define recombination sites. However, this value is consistent with the results from Didelot *et al* obtained by resequencing microarrays, wherein the r/m values among five lineages of *S. enterica* ranged from 0.15 to 2.95 [25].

#### SNPs in the whole genome

With the Mauve program, the whole genome alignment was examined among the seven *S.* Paratyphi A strains. A SNP analysis was performed on the complete allele regions present in all seven strains, and the incomplete allele regions present in two to six strains. SNPs located in the repetitive regions were removed. Finally, we detected 7048 mutations from the complete allele regions and 859 mutations from incomplete allele regions (**Table S4**). Moreover, we categorized these regions into three parts: functional gene regions, pseudogene regions and intergenic regions (**Table 2**). The genome sequences of five newly sequenced strains have not been finished yet, and from the analysis result of the pan-genomic composition of functional genes, we know that genes shared by six strains have a dominant place in the dispensable genes (351 of the 465 dispensable orthologous clusters are shared by six strains), so we think that when the analyses of these genomes are finished, so some SNPs currently belonging to the incomplete allele data would be classified into complete allele data. Consequently, the SNP information in the incomplete allele data is provided as reference data only.

**Table 2:** The SNPs detected in the whole genome of all seven strains.

Region		Incomplete allele data		Complete allele data	
		Mutation Sites	Mutation Rate	Mutation Sites	Mutation Rate
Intergenic Region		249	0.50%	1,496	0.31%
Pseudogene Region		338	1.24%	1,118	0.23%
Functional Region	Nonsynonymous	181	0.18%	3,122	0.13%
	Synonymous	91		1,312	
	dN/dS	0.75		1.06	

From the SNP statistical information in the complete allele data, we know that the intergenic regions have the highest mutation rate, and the functional region has a lower mutation rate than the pseudogenes region. Generally, the relative value of the nonsynonymous rate (dN) to the synonymous rate (dS) is used as an indicator of the selective pressure acting on the protein-coding genes [47]. When the ratio of dN/Ds < 1, it indicates that the sequence suffers from purifying selection (negative selection), while a ratio of dN/dS > 1 indicates that the nonsynonymous mutation is not experiencing strong selection pressure. Using the PAML program, we found that the mean ratio of dN/dS in the functional regions in the complete allele data was 1.06, which is slightly higher than that of *S*. Typhi [48]. This means that the core genome of *S.* Paratyphi A is experiencing more nonsynonymous mutations.Phylogenetic relationships among the seven S. Paratyphi A strains

**Figure 4:**
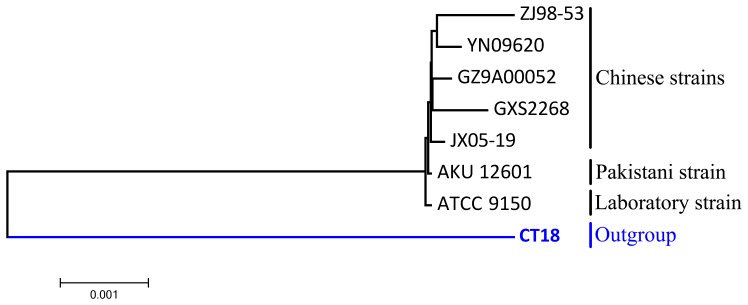
The phylogenetic relationships among all seven *S. *Paratyphi A strains, with *S.* Typhi CT18 used as an outgroup. The phylogenetic tree was based on 1689 core functional genes using the maximum likelihood method. The outgroup strain is marked in blue.

A total of 1689 single-copy core functional genes were utilized to construct the phylogenetic relationships among all seven *S.* Paratyphi A strains, with the corresponding orthologs in *S*. Typhi CT18 used as an outgroup. From **Figure 4**, it is obvious that all five newly sequenced Chinese strains have a closer relationship than ATCC 9150 and AKU_12601. ATCC 9150 is a laboratory strain [23] and differentiated individually from the other six *S.* Paratyphi A strains. AKU_12601 was isolated from a Pakistani paratyphoid patient in Karachi, Pakistan in 2002. In the five newly sequenced strains, JX05-19, a strain isolated from the environment, was separately located from the other four Chinese strains, which were all isolated from patients in the clinic. Strains GXS2268 (isolated in 2007 in Guangxi) and GZ9A00052 (isolated in 2000 in Guizhou), ZJ98-53(isolated in 1998 in Zhejiang) and YN09620 (isolated in 2009 in Yunnan) are closer in the phylogenetic tree, although the isolation dates of each pair of strains covered a 7- to 11-year time span, and the strains were isolated in different sites. GXS2268 and GZ9A00052 were isolated from two neighboring provinces in China, which may partially contribute to their closer phylogenetic relationship, but the isolation sites of ZJ98-53 and YN09620 were spatially distant, separated by several provinces, so the closer phylogenetic relationship may indicate the genetically high conservation of the *S*. Paratyphi A serovars. Our PFGE study demonstrated that the strains from Zhejiang and Guizhou provinces isolated from different years had the same *Xba* I pattern (but different patterns of *Spe* I digestion) [49]. A MLST analysis also showed that a highly conserved clone of *S*. Paratyphi A caused the epidemics in China [50].

#### The “pan-genome of pseudogenes” of S. Paratyphi A

**Figure 5:**
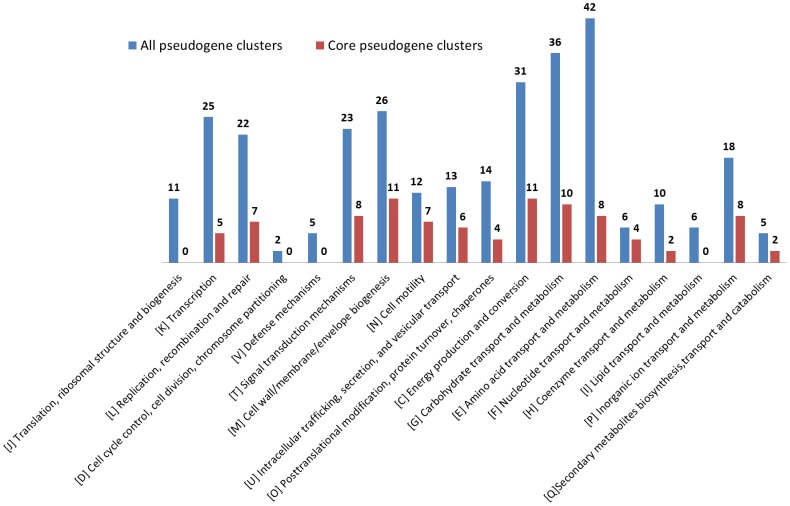
The distribution of pseudogene functions based on the COG classification.

In the pan-genomic analysis of functional genes, all of the functional genes were classified into three groups, the core functional gene clusters, dispensable functional gene clusters and strain-specific functional clusters. To clarify the relationship and distribution of pseudogenes in each strain, all 1646 pseudogenes from the seven strains were also classified into 468 pseudogene clusters according to their original function (**Table S5**). Among the 468 pseudogene clusters, 156 clusters (33.3% of the total pseudogene clusters) were core pseudogene clusters, which were present in all strains. Compared with the core functional gene clusters in all functional gene clusters, the core pseudogene clusters do not have a dominant place in the composition of the pseudogenes. In contrast, the number of strain-specific pseudogene clusters (234 clusters, 50% of the total pseudogene clusters) was slightly larger than the number of core pseudogenes. According to the COG classification, the functions of pseudogene clusters (excluding poorly characterized pseudogenes) were rich in amino acid transport and metabolism, carbohydrate transport and metabolism and cell wall/membrane/envelope biogenesis, while the functions of core pseudogene clusters were rich in energy production and conversion, carbohydrate transport, metabolism, and amino acid transport and metabolism (**Figure 5**).

To understand the features of the pseudogene population quantitatively, we examined the relationship between the pan-genome sizes of pseudogenes or the number of core pseudogene clusters and the number of strains using the same method as was used for the functional genes (**Figure 2B**). From the core pseudogene clusters curve, it is apparent that when up to seven strains were included in the analysis, the decrease in the number of pseudogene core clusters in *S.* Paratyphi A is very slow, and the number of pseudogene core clusters is approaching convergence. From the figure, it is obvious that *S.* Paratyphi A has an open pan-genome of pseudogenes. With regard to the pseudo pan-genome size, although it is an intractable task to find a applicable model for the total pseudogene number in the population, we were able to estimate that about 38 new pseudogene clusters would be identified when another new strain is sequenced.Some functional gggenes are degrading

To elucidate the relationship between pseudogenes and functional genes, all 78 dispensable pseudogene clusters and 234 strain-specific pseudogenes were employed to align the functional gene clusters using their nucleotide sequences. If both the identity and the coverage between a pseudogene and functional gene were over 70%, the pseudogene and functional gene were considered to be homologous sequences. We found that 235 out of the entire 312 pseudogene clusters were homologous to functional gene clusters. Among these 235 pseudogene clusters, 234 pseudogene clusters were homologous to the functional gene clusters shared by one to six strains, while the other one was homologous to the core functional gene cluster.

From the whole genome SNPs analysis, we found that the pseudogene regions in both the complete allele regions and the incomplete allele regions were suffering from a high mutation frequency, so some functional genes which are located in these regions, but not yet inactivated, will likely be inactivated in the future. However, some dispensable functional genes are homologous to current known pseudogenes, so inactivation of these functional genes would not increase the number of total pseudogene clusters. Based on our hypothesis that *S*. Paratyphi A has an open pan-genome of pseudogenes, with more *S*. Paratyphi A strains sequenced, the total pseudogene cluster number would also increase. In this case, when more *S*. Paratyphi A strains are sequenced, many functional genes, which are not homologous to the currently known pseudogenes, would be found to be inactivated in the future sequenced strains. These functional genes that will become inactivated are not only strain-specific genes and dispensable genes (shared by 2 to 6 strains), but also core genes. Once the genes in the core cluster is inactivated, the current core gene cluster would be re-defined as a dispensable cluster or a strain-specific gene cluster so that the increasing number of pseudogenes would lead to a decrease in the core functional cluster. Moreover, in the section of pan-genomic analysis, we inferred that *S*. Paratyphi A strains have a closed pan-genome of functional genes, which means that *S.* Paratyphi A has a deficiency in importing new genes from outside species, and that these strains have a closed gene repertoire. Therefore, it can be concluded that the functional gene number in *S*. Paratyphi A is decreasing and the *S*. Paratyphi A genome is undergoing degradation.

However, it remains unclear whether there are some mechanisms existing in *S*. Paratyphi A which could prevent its genome from becoming overly degraded, like *Mycobacterium leprae* (*M*. *leprae*), which contains 1133 pseudogenes and 1614 functional CDS [51]. There are not enough genomic data currently available for both *S*. Paratyphi A and other species of *Salmonella*, so the question whether *S*. Paratyphi A employs some mechanism to protect its genome from being excessively degraded will require further exploration.

### Conclusions

Based on the genome structure and pan-genomic composition of functional genes, we concluded that the *S.* Paratyphi A genome is extremely conserved. Due to the lack of imported new genes, the entire functional gene pool of *S.* Paratyphi A is closed. From the SNP calling of core functional clusters, we also found signs of frequent recombination events. The pan-genome size and core cluster number, both in functional genes and pseudogenes, made it clear that the sharply increasing number of pseudogene clusters could account for the evolutionary inactivation of functional genes. Furthermore, given the dynamics of the functional gene clusters and pseudogene clusters, we inferred that the *S.* Paratyphi A genome is being degraded.

## Materials and Methods

### Bacterial Strains

*S.* Paratyphi A strains ZJ98-53, GZ9A00052, JX05-19, GXS2268 and YN09620 were respectively isolated from five different high-incidence endemic regions (Zejiang, Guizhou, Jiangxi, Guangxi and Yunnan province) in 1998, 2000, 2005, 2007 and 2009. While JX05-19 was isolated from the environment, the other four strains were isolated from patients in the clinic. The five isolates display different PFGE patterns with XbaI or SpeI digestion, and are all erythromycin and nalidixic acid resistant. The strains were cultured overnight in LB at 37°C with shaking, and chromosomal DNA was prepared using the DNeasy Blood & Tissue Kit (Qiagen Inc) according to the manufacturer’s protocol. The ATCC9150 [23] and AKU-12601 [22] strains were included in our comparative genomic analysis.

### Sequencing and Assembly

The five isolates were sequenced using an Illumina/Solexa Genome Analyzer System according to the manufacturer’s specifications at BGI-Shenzhen with the whole genomic shotgun libraries (insert sizes, ∼500 bp). The raw reads which satisfied one of the following rules were taken as low quality reads: i. there was one or more N’s in the first 20 bases; ii. the percentage of bases with a quality below 10 was higher than 10%; iii. the percentage of bases with a quality below 13 was more than 20%; vi. the average quality of all bases was lower than 20. After removing the low quality reads, 4640879, 3057990, 3068605, 3834202, and 3158039 pairs of high quality pair-end reads (73 nt, 75 nt) were available for the GXS2268, GZ9A00052, JX05-19, YN09620 and ZJ98-53 strains, respectively. The SOAPdenovo [52] and CAP3 [53] software packages were used for the sequence assembly. Finally, the sequences of ZJ98-53, YN09620, GXS2268, JX05-19 and GZ9A00052 were assembled into 24, 24, 25, 27 and 30 scaffolds, respectively.

After assembly, the filtered reads were mapped to the corresponding scaffolds with BWA [54], and improperly mapped reads were removed according to their FLAG values in the SAM file from BWA (reads that obeyed all three of the following rules were considered to be properly mapped reads: both of the mated reads were mapped to the scaffold, they were mapped to different strands, and the distance between their locations on the scaffold was consistent with the insertion length of the library). The false assembly regions on the scaffolds were checked manually with the Tablet visualization program [55] by scanning the low coverage regions on each scaffold.

SNP calling was performed to make sure that the bases on each scaffold were reliably identified. Filtered reads were mapped to the scaffolds with BWA, and potential SNP candidates were identified by Samtools [56]. For each potential SNP candidate, we removed the low quality bases (whose quality was lower than 20) from the corresponding position, and then considered all of the bases (A, T, C and G), whose occurrence was the most frequently observed at the corresponding position in the remaining bases, as the final base on the scaffold.

### Genome Annotation

In order to make CDS prediction easier, all scaffolds for each strain were linked into a pseudochromosome according to the coordinates of ATCC_9150 with a piece of a random sequence. The scaffold linker (NNN NNC ATT CCA TTC ATT AAT TAA TTA ATG AAT GAA TGN NNN N) contains stop and start codons in all six frames, so it could prevent the protein coding genes from extending from one scaffold to the next. Protein coding genes were predicted by the Glimmer3 [57] and gmhmmp programs in GeneMark [58], and then the protein sequences were BLASTed to the Swiss-Prot database, and all the proteins of other bacteria and the protein domains were scanned with InterProScan [59] to remove false positive predicted genes. During the prediction of protein coding genes in the draft genome, some genes could be directly predicted to be present in some strains, while they were missing from the prediction results of other strains. For each *S.* Paratyphi A strain, the entire gene sequences of other *S.* Paratyphi A strains were employed to search the strain’s genome sequence to check whether there were some genes that were present in its genome but missing from the predicted results obtained using the other strain.

For the pseudogene identification, all of the nucleotide sequences of all ORFs were aligned to all functional genes in *Salmonella* strains with the BLAT program [60], and the best five hits were extracted for alignment with Genewise [61]. Genes which obeyed both of the following rules were identified as pseudogenes: 1) there was a frame shift, big indel, or nonsense mutation causing early termination of translation [24]; 2) there were more than three functional genes present in different species or strains, and these genes were consistent in the controversial region in the ORF sequences of the sequenced strains.

In addition, the ribosomal RNA and transfer RNA sequences were predicted by the BLAT program with reference to all the ribosomal RNAs and transfer RNAs present in *Salmonella*.

### Ortholog Identification

Functional orthologous cluster identification was performed by importing protein sequences into the Inparanoid and Multiparanoid software packages using the default parameters (main cutoff: coverage 50% and score 50) [62], [63]. The tree-conflict groups in the Multiparanoid results were extracted and then reclassified manually according to their molecular phylogenetic tree. For the pseudogene orthologous clusters, we gathered all of the pseudogenes present in five strains, and then used the longest pseudogene as the seed pseudogene to BLAT the remaining nucleotide sequences based on the selected cutoff values (identity ≥70% and coverage ≥70%) [37]. Those hit pseudogenes and the seed pseudogene was put in the same cluster, and then the process was carried out for the remaining pseudogenes.

To trace the relationship between functional gene clusters and pseudogene clusters, the nucleotide sequences in each pseudogene cluster were imported into the BLAT program to compare the functional gene clusters’ nucleotide sequence with the cutoff values (identity ≥70% and coverage ≥70%).

### Whole Genome Alignment, SNP Calling and dN/dS Calculation

Whole genome alignments were made by using the Mauve program [64]. From the alignment results, we extracted all of the SNP candidates present in the whole genome. According to the whole genome alignment results, the SNP locations were divided into two parts, which were complete allele regions (the genome regions present in all seven strains) and incomplete allele regions (the genome regions present in two to six strains), and each of these was further split into intergenic regions, pseudo regions, and functional regions. SNPs located in the repetitive regions were excluded. Then, the SNPs were categorized into different classes according to their position within the whole genome. The dN/dS of the functional regions in both the complete allele data and incomplete allele data were calculated with the yn00 program in the PAML [65] package.

For each core functional cluster, all protein sequences in the same cluster were aligned using the ClustalW2 program [66], then the amino acid sequences were replaced with the corresponding codons. Each position was analyzed to detect indel events and mutation events, and every base variation at each position was considered to be a SNP.

### Phylogenetic Analysis

To clarify the phylogenetic relationships between the *S*. Paratyphi A strains, we employed *S.* Typhi CT18 as an outgroup. The core functional clusters with single-copy genes from each strain were utilized as seeds to search the proteome of *S.* Typhi CT18, with coverage ≥90% and identity ≥90%. The hits and the corresponding clusters were taken as the ultimate candidate genes for the phylogenetic analysis. Each candidate gene cluster was aligned separately, and the alignment results were concatenated into one large sequence alignment. A phylogenetic tree was constructed using the phylip software package [67] with the maximum likelihood method.

## Supporting Information

Table S1 New predicted protein coding genes in the two reference strains AKU_12601 and ATCC_9150.
(XLS)

Table S2 Functional gene clusters in the whole seven *S.* Paratyphi A strains.
(XLS)

Table S3 Mutation frequency list in all core functional gene clusters.
(XLS)

Table S4 SNPs list in all the seven *S.* Paratyphi A strains.
(XLS)

Table S5 Pseudogene clusters in the whole seven *S.* Paratyphi A strains.
(XLS)
